# Feature selection using angle modulated simulated Kalman filter for peak classification of EEG signals

**DOI:** 10.1186/s40064-016-3277-z

**Published:** 2016-09-15

**Authors:** Asrul Adam, Zuwairie Ibrahim, Norrima Mokhtar, Mohd Ibrahim Shapiai, Marizan Mubin, Ismail Saad

**Affiliations:** 1 Applied Control and Robotics (ACR) Laboratory, Department of Electrical Engineering, Faculty of Engineering, University of Malaya, 50603 Kuala Lumpur, Malaysia; 2 Faculty of Electrical and Electronic Engineering, Universiti Malaysia Pahang, 26600 Pekan, Pahang Malaysia; 3 Malaysia-Japan International Institute of Technology, Universiti Teknologi Malaysia Kuala Lumpur, Jalan Semarak, 54100 Kuala Lumpur, Malaysia; 4 Artificial Intelligence Research Unit (AiRU), Faculty of Engineering, Universiti Malaysia Sabah, Jalan UMS, 88400 Kota Kinabalu, Sabah Malaysia

**Keywords:** Neural network with random weights (NNRW), Kalman filtering, Simulated Kalman filter (SKF), Electroencephalogram (EEG), Peak detection algorithm, Pattern recognition

## Abstract

In the existing electroencephalogram (EEG) signals peak classification research, the existing models, such as Dumpala, Acir, Liu, and Dingle peak models, employ different set of features. However, all these models may not be able to offer good performance for various applications and it is found to be problem dependent. Therefore, the objective of this study is to combine all the associated features from the existing models before selecting the best combination of features. A new optimization algorithm, namely as angle modulated simulated Kalman filter (AMSKF) will be employed as feature selector. Also, the neural network random weight method is utilized in the proposed AMSKF technique as a classifier. In the conducted experiment, 11,781 samples of peak candidate are employed in this study for the validation purpose. The samples are collected from three different peak event-related EEG signals of 30 healthy subjects; (1) single eye blink, (2) double eye blink, and (3) eye movement signals. The experimental results have shown that the proposed AMSKF feature selector is able to find the best combination of features and performs at par with the existing related studies of epileptic EEG events classification.

## Background

The use of electroencephalogram (EEG) signals for measurements has become a growing interest in research for various applications such as brain-computer interface (Nicolas-Alonso and Gomez-Gil 2012), human–machine interface (Ramli et al. 2015), diagnosing and monitoring epilepsy (Acir 2005), and tracking eye gaze (Adam et al. 2014). Nowadays, the utilization of an advanced processing method makes the EEG signals has efficiently been used in a wide range of applications.

In general, a peak point is defined by a point that holds the highest value located at a specific time and location on EEG signals. A peak point can be observed in EEG signals because of the response of brain on human activities. Such responses of the brain on human activities that triggers a peak in EEG signals are eye movements, epilepsy, and event-related potentials. However, EEG signals are also very sensitive to noises that come from heart bit, EEG electrodes and some movements of the body. The presence of various noises in EEG signals generates a large number of false peaks in the signals and makes the classification of desired peak points difficult. Moreover, this problem could be worse because the amplitude of peaks of the signals is different from one subject to another, which can vary from 600 to 1100 µV (Iwasaki et al. 2005), resulting a high variance value of peak features in data collection.

At present, researchers have used several combinations of peak features based on a time-domain characteristic of the peak in EEG signals (Dumpala et al. 1982; Acir et al. 2005; Acir and Guzelis 2004; Liu et al. 2002; Dingle et al. 1993). Those peak features were obtained from different amplitudes, widths, and slopes. For instance, the peak-to-peak amplitude of the first and second half waves, peak width, ascending peak slopes at the first half wave, and descending peak slope at the second half wave, can be used as the peak features. The peak features are selected to make sure that only relevant features are used for classification. The combinations of the selected features, however, are problem dependent and only efficiently used for a specific application. Furthermore, to properly determine the best and generalized combination of peak features in EEG signals are still open problems for further research.

To avoid the slow learning speed and iteratively learning problems of conventional neural networks learning algorithm (i.e., gradient descent and Levenberg-Marquart), a neural network with random weights (NNRW) is employed as a classifier. The NNRW is a fast, simple, and non-iterative learning algorithm of a single layer feedforward neural network (SLFN). The NNRW was firstly introduced by Schmidt (1992). The network of NNRW consists of three layers that are input, hidden, and output layers. The learning concept of NNRW is that the input weights and the biases at the hidden layer in the network are chosen randomly with a specific interval, whereas the output weights are estimated by the Moore–Penrose generalized inverse method (Rao and Mit 1971). The input weights are assigned randomly between −1 and 1. Also, the biases in the hidden layer are assigned randomly between 0 and 1. Both parameters follow the setup parameters that have been suggested by Cao et al. (2015). A similar concept of NNRW was further developed by Pao and Takefuji (1992), knowingly as random vector functional-link (RVFL) nets. Variations of extended RVFL were introduced to establish the theoretical results of the RVFL concept (Pao et al. 1994; Igelnik and Pao 1995).

Population-based metaheuristic optimization algorithms provide a satisfactory solution in a relatively shorter time. These algorithms are also efficient and effective to solve large and complex real-world problems and can be applied to solve almost any optimization problems (Xiong et al. 2015). A variety population-based metaheuristic optimization algorithms have been invented, such as genetic algorithm (Hooker 1995), simulated annealing (Johnson et al. 1989), particle swarm optimization (Kennedy and Eberhart 1995), ant colony optimization (Dorigo et al. 1996), big bang-big crunch optimization (Erol and Eksin 2006), intelligent water drops algorithm (Shah-Hosseini 2007), honey bee mating optimization (Marinakis et al. 2011), firefly algorithm (Yang 2010b), gravitational search algorithm (Rashedi et al. 2009), harmonic search optimization (Yang 2009), bat algorithm (Yang 2010a), and black hole algorithm (Hatamlou 2013). So far, those optimization algorithms have been already applied as an effective technique for feature selection in various real-world applications such as power system (Ahila et al. 2015), manufacturing (Zhang et al. 2015), and medical (Bababdani and Mousavi 2013; Adam et al. 2014).

Recently, a new metaheuristic optimization algorithm has been introduced by Ibrahim et al. (2015) that is inspired by the state estimation process of Kalman filter. The new optimizer is namely as a simulated Kalman filter (SKF) algorithm. The principle of Kalman filter consists of the following main processes: states prediction, state measurement, and state estimation. In the SKF algorithm, each agent acts as an individual Kalman filter and holds a vector state. Through the prediction, measurement, and estimation state processes, new states are estimated and new locations of agents are updated. The processes are iteratively looped until it reaches the maximum iteration. Regarding the final experimental results by Ibrahim et al. (2015), the SKF algorithm has the capability to find efficiently the most optimal solution and the performance are comparable to gravitational search algorithm and black hole algorithm for unimodal optimization problems. The original SKF algorithm, however, cannot be used for solving discrete optimization problems. To solve this problem, Md Yusof et al. (2016) have introduced an angle modulated SKF (AMSKF) algorithm. Based on the capability of the AMSKF algorithm for solving discrete problems, AMSKF is employed as a feature selection method in this study.

The key contributions of this study are expressed as follows: (1) to employ a recently introduced population-based metaheuristic optimization algorithm for feature selection in EEG signals peak classification using AMSKF, (2) to firstly employ the NNRW into peak detection algorithm for classification and feature selection, (3) to propose a new generalized peak model for EEG signals peak classification based on the features selected by AMSKF, and (4) to apply the proposed method of AMSKF model on epileptic EEG signals. For the benchmarking purpose, four existing peak models are considered. The experimental results show the new combination of peak features that are produced by the proposed AMSKF technique performs better accuracy compared to the NNRW with conventional peak models.

## Data descriptions

### Eye event-related EEG data

The peak candidate data of eye event-related were collected from three different event-related EEG signals that producing peaks. The first peak event-related is labelled as single eye blink signals. The second peak event-related is labelled as double eye blink signals. The third peak event-related is labelled as eye movement signals. The first and second peaks event-related of EEG signals recording were conducted using the g.USBamp biological signals acquisition system. While, the third peak event-related of EEG signals recording were conducted using the g.MOBIlab portable biological signals acquisition system. The scalp electrodes arrangement of the three different signals is placed using the 10–20 international electrode placement system. The sampling frequency for those signals was set to 256 Hz.

The single blink and double blink signals were recorded from F9 channel. The reference electrode was located on the ear. The ground electrode was located on channel AFz. In total, only three electrodes were used. The electrodes from the F9 channels are positioned for detecting EEG peaks associated with the brain response of commanded single and double eye blink. Single means the eye are blinking once while double means the eye are blinking twice. The eyes blink that produces some peaks in the signals on channel F9 is archived as raw data for analysis.

The eye movement signals were recorded from C3 and C4 channels. The channel CZ was used as a reference. The ground electrode was located on FPz channel. In total, only four electrodes were used. The electrodes from the C3 and C4 channels are positioned for detecting EEG peaks associated with the brain response of commanded horizontal eye gaze direction. The eye gaze directions that produce some peaks in the signals on channels C3 and C4 are archived as raw data for analysis.

**Fig. 1:**
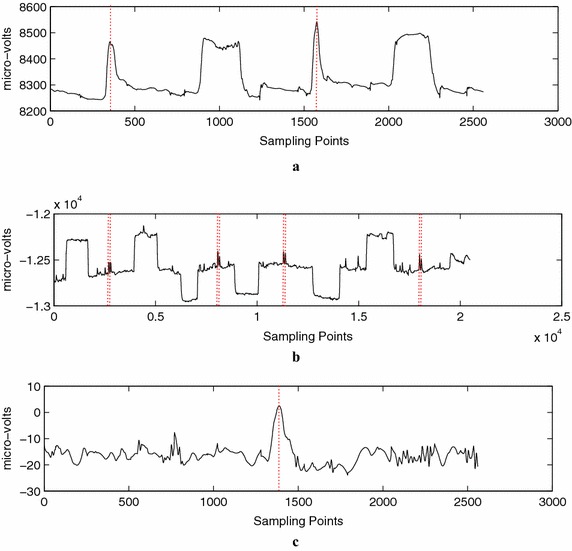
The example of recorded EEG signals: **a** single eye blink (tow peak points per signal), **b** double eye blink (eight peak points per signal), and **c** eye movement (one peak point per signal)

Figure 1a–c shows three different EEG signals that were named as a single eye blink, double eye blink, and eye movement signals. The dotted red vertical lines show the actual peak point location, as manually assigned by a researcher. The descriptions of those EEG signals are tabulated in Table 1.

**Table 1:** Description of the eye event-related EEG signals

Type of signal	No. of signals	No. of sampling points per signal	Length per signal (second)	No. of peaks per signal	Class distribution per signal (peak point/non-peak point)	Total number of (candidate peaks/true peaks/false peaks)
Single eye blink	30	2560	10	2	2/2558	3238/60/3178
Double eye blink	5	20,480	80	8	8/20472	4662/40/4622
Eye movement	40	2560	10	1	1/2559	3881/40/3841
Total EEG data						11,781/140/11,461

The single eye blink signals have 30 signals, 10-s length per signal, 2560 sampling points per signal, and each signal containing two known peak points and various additional signal patterns. The additional signal patterns are the edge transitions which represent the eye movements. The known peak pattern in this signal represents a single eye blink. The peak pattern of a single eye blink is useful as an additional feature for controlling an electric wheelchair (Lin and Yang 2012). The total training and testing sampling points are 38,400 and 38,400, respectively. From the total sampling points, 3238 sampling point locations are identified as the locations of peak candidates, 60 sampling point locations are identified as the locations of true peaks, and 3178 sampling point locations are identified as the locations of false peaks.

The double eye blink signals have five signals, 80-s length per signal, 20,480 sampling points per signal, and each signal containing eight known peak points and some additional signal patterns. The additional signal patterns are the edge transitions that represent the horizontal eye movements. The signals occasionally contain a peak of the single eye blink. The total training and testing sampling points are 51,200 and 51,200, respectively. From the total sampling points, 4662 sampling point locations are identified as the locations of peak candidates, 40 sampling point locations are identified as the locations of true peaks, and 4622 sampling point locations are identified as the locations of false peaks.

Figure 1c shows the eye movement signals. The eye movement signals have 40 signals of C3 and C4 channels, 10-s length per signal, 2560 sampling points per signal, and each signal containing one known actual peak point location. The known peak pattern in this signal represents the horizontal eye gaze direction, either to the left or the right. In total, the data collection of this signal has 40-s length and 102,400 sampling points. From 102,400 sampling points, 3881 candidate peak locations were recognized where the known actual peak point locations are 40 and the remaining sampling points are the known actual non-peak point location.

From the collected raw data of the three EEG signals, 11,781 peak candidate samples with their associated features were archived as EEG data for experiments. From 11,781 peak candidate samples, 140 were assigned as true peaks and the other 11,461 were assigned as false peaks.

### Epileptic EEG data

The second data used in this study is available and published in Bonn University EEG database (Andrzejak et al. 2001). The EEG recording was prepared using standard 10–20 electrode placement system. The datasets have five different sets, which are named as set A, set B, set C, set D, and set E. Each set contains 100 EEG segments that were selected from continuous multi-channel EEG recordings after removing muscle activity or eye movement artifacts. Each EEG segment consists of 4097 sampling points and the duration is about 23.6 s. Sets A and B consist of EEG segments taken from surface EEG recording collected from five healthy subjects. Subjects were relaxed in an awaken state with eyes open (A) and eyes closed (B), respectively. Sets C, D, and E were taken from EEG archive of presurgical diagnosis. Segments in set D were recorded from the epileptogenic zone. Set C is recorded from hippocampal formation of opposite hemisphere of brain. Sets C and D contain only activity measured during epileptic-free intervals. Set E contains only epileptic events. Data is recorded within 128-channel amplifier system and digitized at 173.61 Hz sampling rate and 12 bit A/D resolution. To select the EEG signal of desired band a band-pass filter having a pass band of 0.53–40 Hz (12 dB/oct) was used. In this study, only set A and set E were used. Set A represents as non-epileptic peak events while set E denotes as epileptic peak events.

From the collected EEG raw data of the two sets EEG signals (set A and set E), 20,000 peak candidate samples with their associated features were archived as EEG data for experiments. From 20,000 peak candidate samples, 10,000 were assigned as epileptic peaks event from set E. The other 10,000 were assigned as non-epileptic peaks event from set A. 100 peak candidate samples were randomly selected from each segment of both set. The four-fold cross-validation process is used to produce four groups of EEG data. The class distribution of the peak candidate sample and event is summarized in Table 2.

**Table 2:** Class distribution of the peak candidate sample and event

Class	No. of peak candidate samples	No. of events	Partition of EEG data
Epileptic	10,000	100	Fourfold cross validation
Non-epileptic	10,000	100	
Total	20,000	100	

## Methods

The methods for peak detection consist of three main processes: (1) feature extraction, (2) feature selection, and (3) classification. In feature extraction stage, three-points sliding window method (Dumpala et al. 1982; Billauer 2012) is employed to identify all possible peak candidates. The AMSKF feature selector is used to select the best combination of features for all possible peak candidates. All identified peak candidates with the selected associated features are then classified by the NNRW classifier. The choice of classification method was supported by two reasons: (1) the NNRW provides fast learning speed. (2) The fast learning speed capability in the proposed AMSKF technique can minimize the computational complexity.

### Feature extraction

**Fig. 2:**
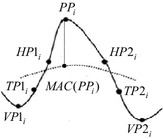
Eight point locations of a peak candidate

So far, to the best of our knowledge, only four models in the time domain analysis have typically been used in various event-related signals for peak classification (e.g., Dumpala et al. 1982; Acir and Guzelis 2004; Liu et al. 2002; Dingle et al. 1993). In general, all existing peak models (i.e., Dumpala, Acir, Liu, and Dingle models) have their associated features. All 16 peak features of the existing models can be calculated using the defined eight parameter points as shown in Fig. 2.

After the *i*th candidate peak point, *PP*_*i*_, and the two associated valley points, $VP1_{i}$ and $VP2_{i}$, are identified using three-points sliding window method (Dumpala et al. 1982; Billauer 2012), the other five parameter points {i.e., the half point at first half wave (*HP*1_*i*_), the half point at second half wave (*HP*2_*i*_), the turning point at first half wave (*TP*1_*i*_), the turning point at second half wave (*TP*2_*i*_), and the moving average curve point [*MAC*(*PP*_*i*_)]} can be identified. For example, the half point at first half wave can be defined as the point located in the middle between the $PP_{i}$ and $VP1_{i}$ while the half point at the second half wave as the point based in the midst between the $PP_{i}$ and $VP2_{i}$. The turning point can be recognized when the slope decreases more than 50 % as compared to the slope of the preceding point. The *MAC(PP*_*i*_) point is located at the intersection between the $PP_{i}$ and *MAC(PP*_*i*_) points.

After all eights parameter points are identified, 16 peak features are then calculated based on the listed equation in Table 3. All peak features can be categorized into three groups, namely amplitude, width, and slope, resulting in five different amplitudes (i.e., *f*_1_, *f*_2_, *f*_3_, *f*_4_, *f*_5_), seven different widths (i.e., *f*_6_, *f*_7_, *f*_8_, *f*_9_, *f*_10_, *f*_11_, *f*_12_), and four different slopes (i.e., *f*_13_, *f*_14_, *f*_15_, *f*_16_). The descriptions of all the 16 features are also explained in Table 3.

**Table 3:** Equations and descriptions of peak features

Peak feature	Feature name	Equation	Description
Amplitudes	Peak-to-peak amplitude of the first half wave	$f_{1} = \left| {x\left( {PP_{i} } \right) - x\left( {VP1_{i} } \right)} \right|$	Amplitude between the magnitude of peak and the magnitude of valley at the first half wave
	Peak-to-peak amplitude of the second half wave	$f_{2} = \left| {x\left( {PP_{i} } \right) - x\left( {VP2_{i} } \right)} \right|$	Amplitude between the magnitude of peak and the magnitude of valley of the second half wave
	Turning point amplitude of the first half wave	$f_{3} = \left| {x\left( {PP_{i} } \right) - x\left( {TP1_{i} } \right)} \right|$	Amplitude between the magnitude of peak and the magnitude of turning point at the first half wave
	Turning point amplitude at the second half wave	$f_{4} = \left| {x\left( {PP_{i} } \right) - x\left( {TP2_{i} } \right)} \right|$	Amplitude between the magnitude of peak and the magnitude of turning point at the second half wave
	Moving average amplitude	$f_{5} = \left| {x\left( {PP_{i} } \right) - MAC\left( {PP_{i} } \right)} \right|$	Amplitude between the magnitude of peak and the magnitude of moving average
Widths	Peak width	$f_{6} = \left| {VP1_{i} - VP2_{i} } \right|$	Width between valley point of first half point and valley point at second half wave
	First half wave width	$f_{7} = \left| {PP_{i} - VP1_{i} } \right|$	Width between peak point and valley point at first half wave
	Second half wave width	$f_{8} = \left| {PP_{i} - VP2_{i} } \right|$	Width between peak point and valley point of second half wave
	Turning point width	$f_{9} = \left| {TP1_{i} - TP2_{i} } \right|$	Width between turning point at first half wave and turning point at the second half wave
	First half wave turning point width	$f_{10} = \left| {PP_{i} - TP1_{i} } \right|$	Width between turning point at first half wave and peak point
	Second half wave Turning point width	$f_{11} = \left| {PP_{i} - TP2_{i} } \right|$	Width between turning point at second half wave and peak point
	FWHM	$f_{12} = \left| {HP1_{i} - HP2_{i} } \right|$	Width between half point of first half wave and half point of second half wave
Slopes	Peak slope at the first half wave	$f_{13} = \left| {\frac{{x\left( {PP_{i} } \right) - x\left( {VP1_{i} } \right)}}{{PP_{i} - VP1_{i} }}} \right|$	Slope between a peak point and valley point at the first half wave
	Peak slope at the second half wave	$f_{14} = \left| {\frac{{x\left( {PP_{i} } \right) - x\left( {VP2_{i} } \right)}}{{PP_{i} - VP2_{i} }}} \right|$	Slope between a peak point and valley point at the second half wave
	Turning point slope at the first half wave	$f_{15} = \left| {\frac{{x\left( {PP_{i} } \right) - x\left( {TP1_{i} } \right)}}{{PP_{i} - TP1_{i} }}} \right|$	The slope between peak point and turning point at the first half wave
	Turning point slope at the second half wave	$f_{16} = \left| {\frac{{x\left( {PP_{i} } \right) - x\left( {TP2_{i} } \right)}}{{PP_{i} - TP2_{i} }}} \right|$	The slope between peak point and turning point at the second half wave

Table 4 presents the list of different peak models with their associated features. The Dingle model is produced by four features: *f*_5_, *f*_6_, *f*_13_, and *f*_14_. The associated features of Dumpala model are denoted as *f*_1_, *f*_6_, *f*_13_, and *f*_14_. Acir model consists of six features: *f*_1_, *f*_2_, *f*_7_, *f*_8_, *f*_13_, and *f*_14_. The considerably more complex model of Liu et al. (2002) entails 11 features: *f*_1_, *f*_2_, *f*_3_, *f*_4_, *f*_6_, *f*_9_, *f*_12_, *f*_12_, *f*_14_, *f*_15_, and *f*_16_.

**Table 4:** List of different peak models with their associated features

Peak models	Set of features	Number of features
Dingle	*f*_5_, *f*_6_, *f*_13_, *f*_14_	4
Dumpala	*f*_1_, *f*_6_, *f*_13_, *f*_14_	4
Acir	*f*_1_, *f*_2_, *f*_7_, *f*_8_, *f*_13_, *f*_14_	6
Liu	*f*_1_, *f*_2_, *f*_3_, *f*_4_, *f*_6_, *f*_9_, *f*_12_, *f*_13_, *f*_14_, *f*_15_, *f*_16_	11

### Neural network with random weights (NNRW) classifier

The NNRW classifier has recently gained attention as a fast learning and generalized technique for classification (Cao et al. 2016; Lang et al. 2015). The fundamental aspect of this method is that the NNRW can be represented as a linear system (Schmidt 1992). The linear system of NNRW is mathematically modeled as $H\beta = T$ where *β* is the *L* × *m* matrix of output weights and *T* is the *N* × *m* matrix of target outputs. *m* is the number of output neurons. The *β* and *T* matrixes are denoted as

1 $$\beta = \left[ {\begin{array}{*{20}c} {\beta_{1}^{\rm T} } \\ \vdots \\ {\beta_{L}^{\rm T} } \\ \end{array} } \right]_{L \times m}$$

and

2 $$T = \left[ {\begin{array}{*{20}c} {t_{1}^{\rm T} } \\ \vdots \\ {t_{N}^{\rm T} } \\ \end{array} } \right]_{N \times m} ,$$

respectively. The output function of NNRW classifier of a given unknown sample, *x* can be mathematically described as $fc(x) = h(x)\beta$. The output matrix of the hidden layer, *H*, is calculated as follows:

3 $$H = \left[ {\begin{array}{*{20}c} {h(x_{1} )} \\ \vdots \\ {h(x_{N} )} \\ \end{array} } \right] = \left[ {\begin{array}{*{20}c} {g\left( {\sum\nolimits_{i = 1}^{d} {a_{i1} x_{1i} + b_{1} } } \right)} & \cdots & {g\left( {\sum\nolimits_{i = 1}^{d} {a_{iL} x_{1i} + b_{L} } } \right)} \\ \vdots & \ddots & \vdots \\ {g\left( {\sum\nolimits_{i = 1}^{d} {a_{i1} x_{Ni} + b_{1} } } \right)} & \cdots & {g\left( {\sum\nolimits_{i = 1}^{d} {a_{iL} x_{Ni} + b_{L} } } \right)} \\ \end{array} } \right]_{N \times L}$$

where *g* is an activation function of the hidden neuron, *x* is the *N* × *L* matrix of inputs, *a* is the *d* × *L* matrix of random input weights, *b* is the 1 × *L* matrix of random biases in the hidden layer, *N* is an arbitrary distinct sample, *L* is the number of hidden neurons (*L* = 1000 in this study), and *d* is the number of inputs (where *d* depends on the number of the selected features in this study). The *i*th column of *H* is the output of the *i*th hidden neuron with respect to inputs *x*_1_, *x*_2_, until *x*_*d*_. The sigmoidal function $g(x) = {1 \mathord{\left/ {\vphantom {1 {(1 + e^{ - x} )}}} \right. \kern-0pt} {(1 + e^{ - x} )}}$ was used in this study as an activation function in the hidden layer for normalization while a linear function is located inside the neuron in the output layer.

To find the least square solution, *β* of the linear system, $H\beta = T$, the minimum-norm least-squares solution is computed as follows:

4 $$\left\| {H\left( {a_{1} , \ldots ,a_{L} ,b_{1} , \ldots ,b_{L} } \right)\beta - T} \right\| = \mathop {\hbox{min} }\limits_{\beta } \left\| {H\left( {a_{1} , \ldots ,a_{L} ,b_{1} , \ldots ,b_{L} } \right)\beta - T} \right\|$$

It is well known that the smallest norm least-squares solution of Eq. (4) is

5 $$\beta = (H^{\rm T} H)^{ - 1} H^{\rm T} T = H^{ + } T$$

where *H*^+^ is the Moore–Penrose pseudo-inverse of *H*. The summary of the training stages of the NNRW classifier is listed as follows:

- *Stage 1* Assign randomly the input weights, *a*_*i*_ and biases in the hidden neurons, *b*_*i*_.
- *Stage 2* Calculate the output matrix of the hidden layer, *H*.
- *Stage 3* Calculate the output weights, $\beta = H^{ + } T$.

In the output layer, two neurons are used in the network to classify the output into two classes (output): class 1 and class 0. For two classes (*m* > 1), the predicted class label is the *i*th number of the output neurons which the maximum value of output neuron. The predicted class label of a given unknown sample *x* is defined as follows.

6 $$label(x) = \mathop {\arg \hbox{max} fc_{i} (x)}\limits_{{i \in \left\{ {1, \ldots ,m} \right\}}}$$

The performance of the classifier is evaluated using a four-fold cross-validation process. The four-fold cross-validation accuracy of the classifier is computed using *Gmean* (Guo et al. 2008). The *Gmean* is calculated as follows:

7 $$TPR = \frac{TP}{TP + FN}$$

8 $$TNR = \frac{TN}{TN + FP}$$

9 $$Gmean = \sqrt {TPR \times TNR}$$

where any true peak (*TP*) is the correctly detected apex point of a peak candidate, a true non-peak (*TN*) is any correctly detected non-peak point of a peak candidate, a false peak (*FP*) is an incorrectly designated non-peak point of a peak candidate, a false non-peak (*FN*) is any incorrectly detected true peak point of peak candidate, *TPR* is the true peak rate, and *TNR* is the true non-peak rate.

### Simulated Kalman filter (SKF) for continuous optimization problems

**Fig. 3:**
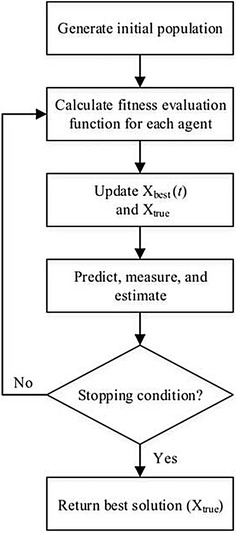
The simulated Kalman filter (SKF) algorithm

The SKF algorithm (Ibrahim et al. 2015) was originally invented for solving continuous optimization problems. The algorithm follows several steps as shown in Fig. 3: (1) generate an initial population, (2) calculation of the fitness evaluation function for each agent, (3) update the best fitness value among agents at every iteration (X_best_) and the best solution compared to the current X_best_ (X_true_), (4) perform state prediction, measurement, and estimation, and (5) perform termination based on a stopping criterion.

In the initialization step, several initial SKF parameters such as the initial value of error covariance estimate, *P*(0), the process noise value, *Q*, and the measurement noise value, *R*, are initialized. Further settings, such as, the number of *n* agents and a maximum number of iterations, $t_{\hbox{max} }$, are also determined. The states values of each agent are given randomly within a specific interval.

Next, the fitness evaluation function is computed to obtain initial solutions for every agent. The best fitness value among each agent at every iteration *t*, X_best_(*t*) can be either in the maximization problem, $\max_{i \in \,1, \ldots ,n} fit\left( {(X(t)} \right)$ or minimization problem $\min_{i \in \,1, \ldots ,n} fit\left( {(X(t)} \right).$

The X_best_(*t*) value at every iteration *t* is compared and the best among the X_best_(*t*) value, which is *X*_true_ is updated. For a maximization problem, X_true_ is only updated when X_best_(*t*) at current iteration is greater than X_true_. Whereas, for a minimization problem, X_true_ is only updated when X_best_(*t*) at current iteration is lower than X_true_.

**Fig. 4:**
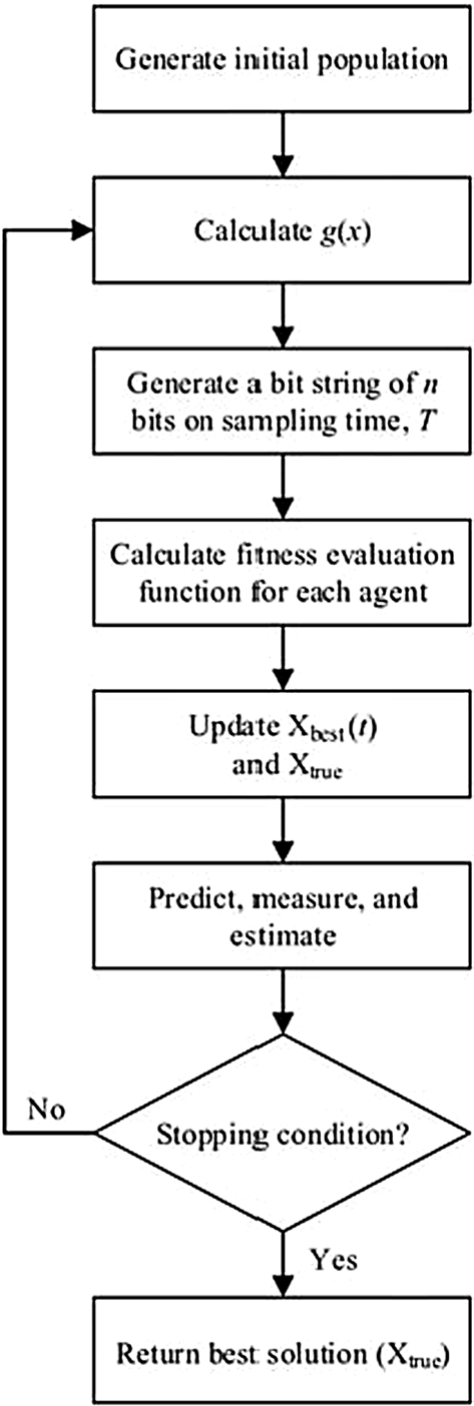
The angle modulated simulated Kalman filter (AMSKF) algorithm

Referring to Fig. 4, the next following steps including the state prediction, measurement, and estimation. The state prediction follows the following equations:

10 $$X_{i} \left( {t|t - 1} \right) = X_{i} \left( {t - 1} \right)$$

11 $$P\left( {t|t - 1} \right) = P\left( {t - 1} \right) + Q$$

where, $X_{i} \left( {t - 1} \right)$ and $X_{i} \left( {t|t - 1} \right)$ are the previous state and transition state, respectively. $P\left( {t|t - 1} \right)$ and $P\left( {t - 1} \right)$ are previous error covariant estimate and transition error covariant estimate, respectively.

In the state measurement step, the following equation, $Z_{i} (t)$, is used, which gives some feedbacks to the estimation process.

12 $$Z_{t} (t) = X_{i} \left( {t|t - 1} \right) + \sin \left( {rand \times 2\pi } \right) \times \left| {X_{i} \left( {t|t - 1} \right) - X_{true} } \right|$$

In Eq. (12), the $\sin \left( {rand \times 2\pi } \right)$ term offers the stochastic element of SKF algorithm which having a random probability distribution to the measurement value and $rand$ is a uniformly distributed random number in the range of [0 1].

Next, the Kalman gain, *K*(*t*), is computed based on the calculated value of the transition error covariant estimate, $P\left( {t|t - 1} \right)$ and the measurement noise value, *R*. The equation of *K*(*t*) is given as follows.

13 $$K(t) = \frac{{P\left( {t|t - 1} \right)}}{{P\left( {t|t - 1} \right) + R}}$$

Here, the equation for estimating the next state, $X_{i} (t)$, is given in Eq. (14) and the error covariant is updated based on Eq. (15). Finally, the processes are iteratively looped until the maximum number of iteration is reached.

14 $$X_{i} (t) = X_{i} \left( {t|t - 1} \right) + K(t) \times \left( {Z_{i} (t) - X_{i} \left( {t|t - 1} \right)} \right)$$

15 $$P(t) = \left( {1 - K(t)} \right) \times P\left( {t|t - 1} \right)$$

### Angle modulated simulated Kalman filter (AMSKF) for discrete optimization problems

For solving discrete optimization problems, the angle modulated concept is embedded into SKF algorithm (Md Yusof et al. 2016). Referring to Fig. 4, additional two steps of the angle modulated into SKF are described as follows. After the initialization step, the continuous signals, *g*(*x*) with four coefficient parameters (*a*, *b*, *c,* and *d*) are generated for each agent. So, the state of the *i*th agent in a population at iteration *t* is denoted as $X_{i} (t) = \left\{ {a_{i} ,b_{i} ,c_{i} ,d_{i} } \right\}$. As mentioned before, the state values which are *a*, *b*, *c*, and *d* are given randomly in an initial stage. The function *g*(*x*) with the four coefficient parameters is defined as follows,

16 $$g(x) = \sin \left( {2\pi (x - a) \times b \times \cos \left( {2\pi (x - a) \times c} \right)} \right) + d$$

**Fig. 5:**
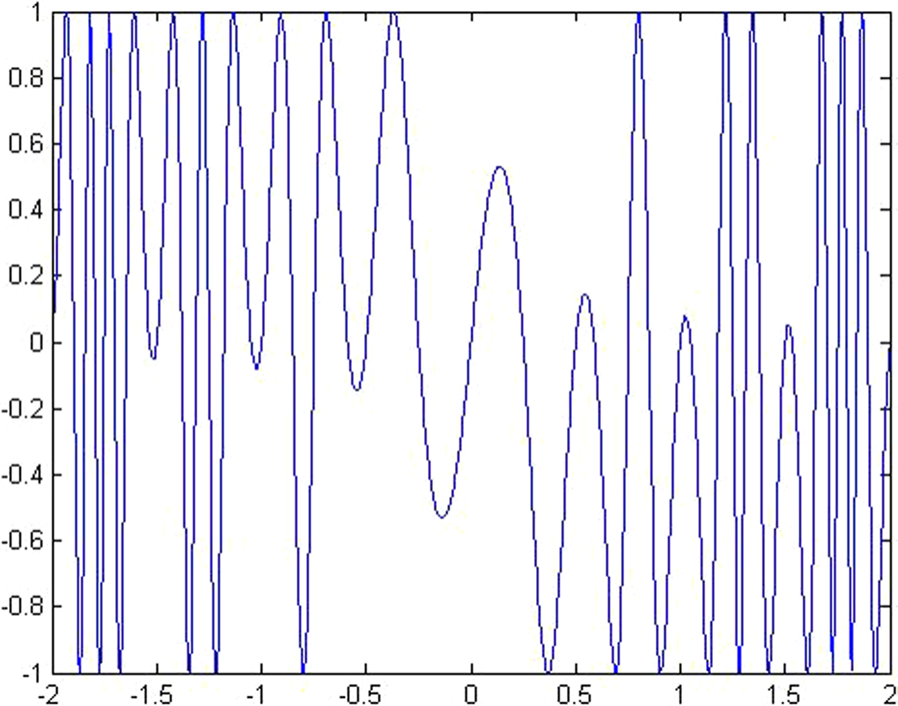
An example of *g*(*x*) function with *a* = 0, *b* = 1, *c* = 1, and *d* = 0

An example plot of function, *g*(*x*) for the case of *a* = 0, *b* = 1, *c* = 1, and *d* = 0 is given in Fig. 5. From the signals, the sampling time, *T*, is chosen to generate a bit string of length *n* in the next step. The bit 1 is generated when g(x) value is greater than 0 while, the bit 0 is generated when *g*(*x*) value is lower than 0. The length of the bit string depends on the given problem. For example, if the length of the full feature set is 100, so the length of the bit string is 100. The generated bit string of each agent is employed to calculate the fitness value for each agent. Then, AMSKF follows similar steps as SKF until it returns the final solution. Using the angle modulated approach, the AMSKF algorithm only tunes the four coefficient parameters for getting the best solution.

## The proposed AMSKF feature selection algorithm

**Fig. 6:**
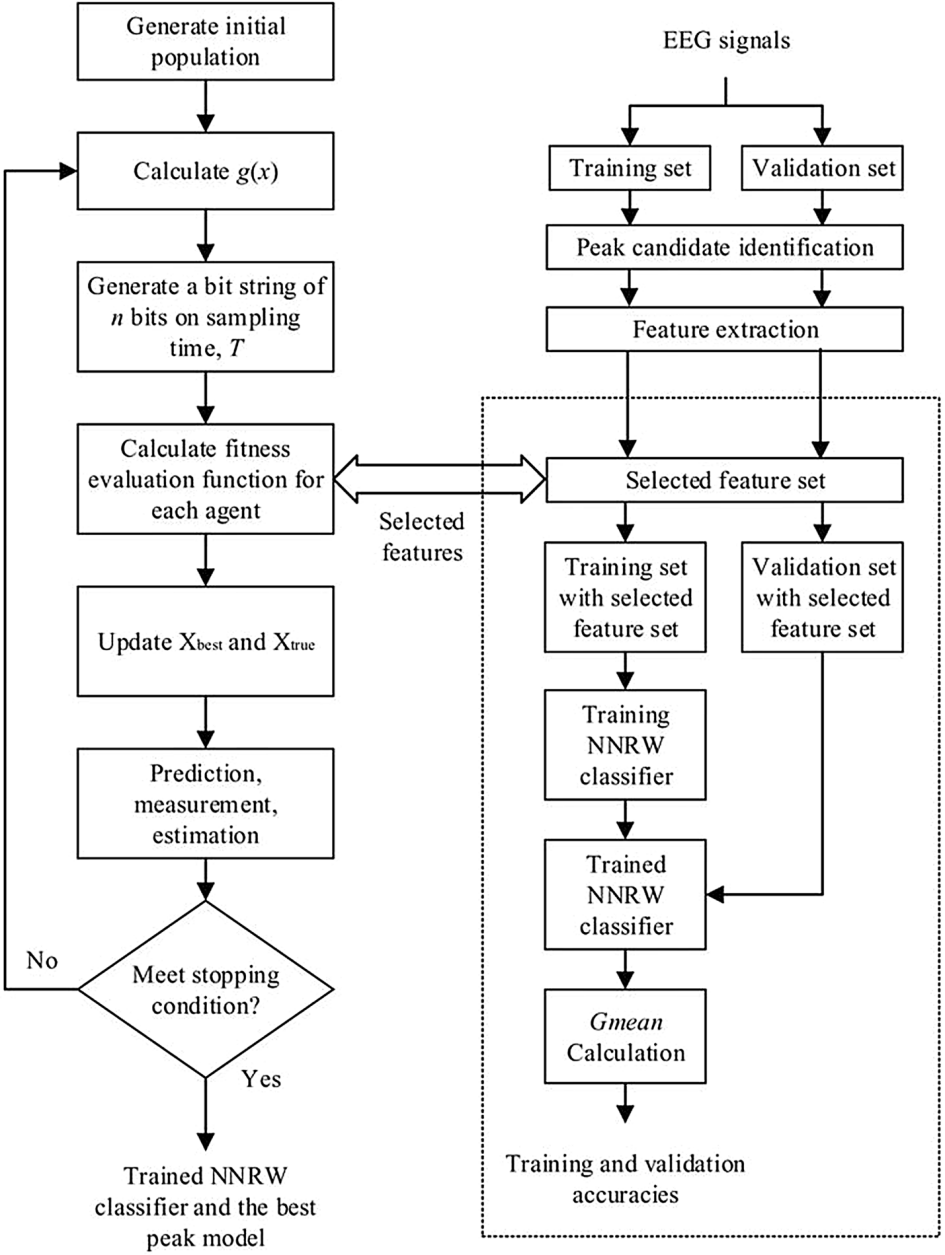
Flowchart of the proposed AMSKF feature selection algorithm

The proposed feature selection algorithm for EEG signals peak detection is based on AMSKF algorithm. Also, the NNRW classifier is employed for peak classification. The combination of both methods is illustrated in the flowchart as shown in Fig. 6.

From Fig. 6, the proposed AMSKF technique begins with initialization of a population and then calculation of a *g*(*x*) function. The maximum number of iteration was set to 500 and the number of agents was set to 10. The initial value of the error covariance estimate, *P*, process noise value, *Q*, and measurement noise value, *R*, are 10,000, 0.5, and 0.5, respectively. To employ AMSKF algorithm for feature selection in EEG peak classification, a total of 16-bit string is generated since the selection of one feature is determined by one-bit value. If AMSKF assigns bit value 1 to an *i*th feature, the *i*th feature is selected. Otherwise, the *i*th feature is not selected.

In the calculation process of the fitness evaluation function, the selected features are used to prepare the training and validation sets, as shown in Fig. 6. To calculate the fitness evaluation function, at first, the classifier has to be trained by the given training data. Then, the trained classifier is tested using the validation set. The detection performance of the training and validation sets are computed based on *Gmean* (Guo et al. 2008). The *Gmean* of validation set is set as fitness value for AMSKF algorithm.

In Fig. 6, after fitness value is calculated, the process continues to the next following processes; update X_best_ (*t*) and X_true_, state measurement, state prediction, and state estimation. Next, new 16 bits solutions are determined and those processes are looped until maximum iteration is reached. Finally, the best peak model associated with the NNRW was obtained.

## Experimental results and discussions

In this section, three main experiments were conducted. The first experiment aimed to investigate the classification performance of the individual NNRW under various number of hidden neurons. This experiment was also evaluated the performance of the individual NNRW over the four existing peak models. The optimum number of hidden neurons was selected to perform the experiment of the proposed AMSKF technique. The second experiment was assigned to study the search capability of the proposed AMSKF technique to find the best combination of peak features. The first and second experiments were conducted on eye event-related EEG data. The third experiment was conducted to apply the best combination of peak features on epileptic EEG classification events application.

### Performance of NNRW under various number of hidden neurons

One advantage of the NNRW classifier is that the learning algorithm is less difficult than other conventional neural network classifier (i.e., gradient descent, Levenberg-Marquart, and particle swarm optimization-based learning algorithms). So that, with an enormous number of hidden neurons is possible to perform using the NNRW classifier. However, the optimal number of neurons of the NNRW classifier is required to be firstly identified for offering better generalization ability of the NNRW classifier. To find the optimal number of hidden neuron, an experiment is executed by varying the number of hidden neuron from 100 to 1200 in steps of 100.

To prepare the experiment data of the individual NNRW classifier, the EEG dataset are randomly divided into four groups, equally distributes the two-class ratio, by four-fold cross-validation process. Every group alternately assigned as the testing set and the other three groups are combined to be a training set. The mean value of testing results from the four groups is calculated. This experiment is repeated 30 times, so that the mean of the training and testing results can be measured as shown in Table 5.

**Table 5:** Classification accuracy results for NNRW classifier under different number of hidden neurons on eye event-related EEG data

Peak model	Result	No. of hidden neurons											
		100	200	300	400	500	600	700	800	900	1000	1100	1200
Dumpala	Train	5.15	30.1	43.61	53.39	60.26	66.51	71.27	75.55	78.63	80.86	82.96	84.54
	Test	1.09	15.77	24.83	31.75	38.09	42.12	45.31	48.17	49.37	51.46	52.9	53.87
Acir	Train	37.69	48.95	53.37	56.87	59.82	63.27	66.41	70.06	73.69	76.3	79.38	81.73
	Test	34.46	44.05	45.11	46.67	47.74	48.55	49.3	50.2	51.86	52.16	51.67	52.91
Liu	Train	35.61	48.54	54.83	60.38	65.41	69.09	71.94	73.99	75.52	77.18	78.62	80.16
	Test	29.18	38.76	41.4	42.97	45.25	46.34	48.07	47.94	48.85	48.19	48.57	48.91
Dingle	Train	0	6.19	19	31.13	41.89	49.91	57.07	61.96	68.14	71.39	75.12	77.22
	Test	0	1.55	6.48	15.97	21.97	25.81	32.34	34.78	38.31	40.13	43.65	45.26

**Fig. 7:**
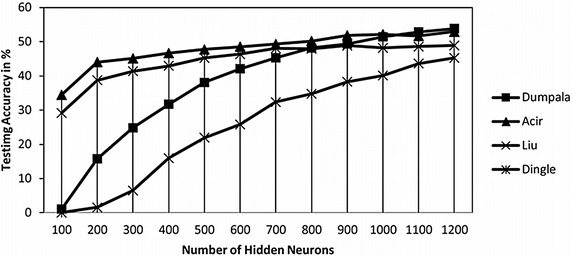
Variation of testing accuracy of NNRW classifier with respect to number of hidden neurons on eye event-related EEG data

The variation of testing accuracy with respect to a different number of hidden neurons is graphically illustrated in Fig. 7. Referring to Fig. 3, the testing accuracy of all four peak models increased up to 1200 neurons. Three peak models (e.g., Dumpala, Acir, and Liu models) except Dingle model offer the optimal accuracy when the numbers of hidden neurons are between 900 and 1200. Hence, the number of hidden neurons for our experiment was set to 1000. The final results in Fig. 7 indicate that the selection of the best combination features is necessary for providing the best and generalizes performance in EEG signals peak classification.

### Experimental results for AMSKF feature selection algorithm

To prepare the experiment data of the proposed AMSKF feature selection algorithm, the four-fold cross-validation process is used to produce four groups of EEG data: each group consists of training and testing sets. Next, the training set is randomly divided into two: training and validation sets. Both datasets are equally distributed to the two-class ratio. The ratio size of training and validation was set to 0.5:0.5. The testing set is utilized as unseen EEG data. After all four groups are evaluated by the algorithm, the maximum value of testing results from the four groups is measured and the best peak model is recorded. This entire four-fold cross validation process is repeated 30 times to obtain the final statistical results (e.g., average, maximum, minimum, and standard deviation) for this experiment.

Table 6 shows the 30 independent runs experimental results of the proposed AMSKF feature selection algorithm using the EEG data that is collected from the three recorded EEG signals (i.e., single eye blink, double eye blink, and eye movement signals). Table 6 gives the best peak model with the highest training, validation, and testing accuracies for the NNRW classifier at every run. In this experiment, the best-generalized peak model is chosen based on the maximum accuracy of testing data over 30 runs.

**Table 6:** Best testing results over 30 runs using the proposed AMSKF feature selection algorithm on eye event-related EEG data

Run	Training (%)	Validation (%)	Testing (%)	Best peak model														Feature subset length
1	87.52	63.88	69.19	1	3	4	6	7	8	9	10	11	12	13	14	15	16	14
2	90.14	63.92	62.89	1	2	3	4	5	6	7	8	9	15	16				11
*3*	95.12	61.30	55.78	1	2	3	4	5	6	7	8	9	10	11	16			12
*4*	*91.77*	*61.68*	*72.71*	*1*	*2*	*7*	*8*	*9*	*10*	*11*	*12*	*13*	*14*	*15*				*11*
5	78.33	65.99	56.51	13	14	15	16											4
6	89.44	71.36	62.21	3	6	7												3
7	93.81	67.50	66.78	1	2	8	9	10	11	12	13	14	15	16				11
8	96.61	67.19	60.02	1	5	9	13											4
9	94.65	64.64	66.50	1	2	14	15											4
10	92.20	60.68	57.87	2	3	8	9	10	13	14								7
11	95.74	66.54	62.55	1	11	12	15											4
12	82.57	65.36	61.47	12	13	14	15	16										5
13	92.20	71.06	64.64	1	2	5	13	14	15	16								7
14	91.50	71.13	59.16	3	6	14												3
15	89.44	58.06	60.60	1	2	3	7	8	10	11	13	15	16					10
16	88.19	65.65	60.32	1	2	5	6	7	8	9	10	13	14	15	16			12
17	90.83	70.24	55.20	1	2													2
18	86.92	67.34	60.51	1	2	5	6	7	8	9	10	13	14	15				11
19	95.24	62.63	61.98	1	2	3	4											4
20	88.80	68.93	66.51	1	2	3	15	16										5
21	85.54	66.92	61.66	9	10	11	12	13	14	15	16							8
22	94.15	66.02	57.85	1	3	4	7	9	11	14	16							8
23	82.12	62.33	61.34	12	13	14	15	16										5
24	95.59	65.14	62.30	1	2	3	9	10										5
25	83.67	68.40	62.37	1	2													2
26	92.08	66.54	61.75	3	9	15	16											4
27	80.18	63.01	61.96	14	15	16												3
28	94.15	66.95	52.96	1	10	11	12	13	14									6
29	87.60	60.47	63.47	12	13	14	15	16										5
30	89.92	71.94	62.34	3	4													2

The best-generalized peak model based on the maximum accuracy of testing data over 30 runs was marked with the italic font

In Table 6, it is found that the feature set of the best peak model is *f*_1_, *f*_2_, *f*_7_, *f*_8_, *f*_9_, *f*_10_, *f*_11_, *f*_12_, *f*_13_, *f*_14_, and *f*_15_, with 72.7 % of testing accuracy. From those associated features, two of features are peak amplitudes (e.g., *f*_1_ and *f*_2_), six of features are peak widths (e.g., *f*_7_, *f*_8_, *f*_9_, *f*_10_, *f*_11_, and *f*_12_), and three of features are peak slopes (e.g., *f*_13_, *f*_14_, and *f*_15_). For overall of testing accuracy, the average, maximum, minimum, and STDEV over 30 runs are 61.7, 72.7, 53, and 4.1 %, respectively.

The results in Table 6 show that the higher value of fitness of validation set cannot produce the best classification accuracy of testing set as expected. Also, the feature set that contain lower feature subset length cannot give better performance. These results have exhibited that the peak event-related EEG signals are very problem dependant.

**Fig. 8:**
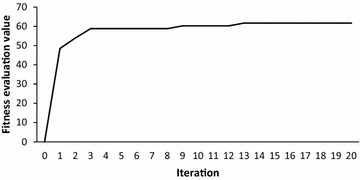
Example of a convergence curve of AMSKF on eye event-related EEG data

In this experiment, the proposed AMSKF algorithm was iteratively executed with maximum 500 iterations. To observe the result of convergence of the proposed AMSKF, one example is taken from this experiment, as illustrated in Fig. 8. From Fig. 8, it can be seen that the AMSKF algorithm can reach convergence within 20 iterations.

To evaluate the effectiveness of the proposed algorithm and the selected best combination of features, some comparisons are performed regarding percentage of the testing classification accuracy between the results of the existing four peak detection models and with the proposed AMSKF model. The comparison results are comparatively presented in Table 7. For a fair performance evaluation, the four existing peak models with their associated features are performed using the similar parameters setting of the NNRW of the proposed AMSKF technique.

**Table 7:** Comparison of the classification accuracy between the existing models and the best combination of features that produced by AMSKF technique on eye event-related EEG data

Peak model	Feature subset length	Selected features	Training accuracy (%)	Testing accuracy (%)
Dumpala	4	*f*_1_, *f*_6_, *f*_13_, *f*_14_	80.9	51.5
Acir	6	*f*_1_, *f*_2_, *f*_7_, *f*_8_, *f*_13_, *f*_14_	76.3	52.2
Liu	11	*f*_1_, *f*_2_, *f*_3_, *f*_4_, *f*_6_, *f*_9_, *f*_12_, *f*_13_, *f*_14_, *f*_15_, *f*_16_	77.2	48.2
Dingle	4	*f*_5_, *f*_6_, *f*_13_, *f*_14_	71.4	40.1
AMSKF (proposed work)	11	*f*_1_, *f*_2_, *f*_7_, *f*_8_, *f*_9_, *f*_10_, *f*_11_, *f*_12_, *f*_13_, *f*_14_, *f*_15_	91.8	72.7

The experimental results in Table 6 are obtained from the experiment in “Performance of NNRW under various number of hidden neurons” section, with the hidden neuron of the NNRW is 1000. The performance of the best combination of features is taken from the maximum testing accuracy in Table 6. As seen from Table 7, the performance of the best combination of features that are produced by AMSKF algorithm exceeds the performance of the other existing four models.

In Table 7, it can be seen that there is a large different value between training and testing accuracies. The proposed method of the AMSKF model has only achieved 73 % of testing accuracy. In this study, the ratio between true peak and false peak is 140:11,461. This means the dataset has extremely imbalanced dataset ratio. In this case, the conventional NNRW classifier may fail to offer high accuracy of performance for imbalanced dataset problem. Other contributing factor is the collected EEG data is affected by various noises and the peak features have a large different value from one subject to another subject. This factor is the cause to the high variation of peak features. The consequent of this factor is that the NNRW classifier may fail to correctly classify the true peak and false peak.

The results of the peak models are further analyzed by using nonparametric Friedman statistical analysis. The statistical analysis is required to demonstrate the significant difference in testing accuracy in terms of average value for the five models. The experiments are conducted based on statistical procedures designed especially for multiple *N* × *N* comparisons with five models executed in the KEEL data mining system (Alcala-Fdez et al. 2009).

Table 8 shows the average ranking of Friedman’s test of the Dumpala, Acir, Liu, Dingle, and AMSKF models. The statistical results show that the lowest average ranking is obtained by AMSKF model that represents ranking first among the five models for EEG data. While, the NNRW with Acir model ranking second, the NNRW with Dumpala model ranking third, the NNRW with Liu model ranking fourth, and the NNRW with Dingle model ranking fifth.

**Table 8:** The average ranking of the Dumpala, Acir, Liu, Dingle, and AMSKF, achieved by Friedman

Peak model	Average ranking	Rank
AMSKF (this work)	1.1	1
NNRW (Acir)	2.533	2
NNRW (Dumpala)	2.733	3
NNRW (Liu)	3.767	4
NNRW (Dingle)	4.867	5
Statistic	95.6533	
*p* value	6.693E−11	

Next, *p* values for unadjusted values and adjusted p values for Nemenyi, Holm’s, Shaffer, and Bergmann-Hommel test for *N* × *N* comparisons for all possible ten pairs of model with the peak models are presented in Table 9. The *p* values below 0.05 represent that the particular peak model differ significantly in testing accuracy. The *p* values below 0.05 were marked with the italic font.

**Table 9:** Adjusted p value for *N* × *N* comparisons of peak models over 30runs

Peak model versus peak model	pUnadj	pNeme	pHolm	pShaf	pBerg
Dingle versus AMSKF	*0*	*0*	*0*	*0*	*0*
Liu versus AMSKF	*0*	*0*	*0*	*0*	*0*
Acir versus Dingle	*0*	*0*	*0*	*0*	*0*
Dumpala versus Dingle	*0*	*0.000002*	*0.000001*	*0.000001*	*0.000001*
Dumpala versus AMSKF	*0.000063*	*0.000631*	*0.000379*	*0.000379*	*0.000252*
Acir versus AMSKF	*0.000447*	*0.004465*	*0.002233*	*0.001786*	*0.000893*
Acir versus Liu	*0.002519*	*0.025191*	*0.010076*	*0.010076*	*0.007557*
Liu versus Dingle	*0.007051*	0.070507	*0.021152*	*0.021152*	*0.014101*
Dumpala versus Liu	*0.011369*	0.113693	*0.022739*	*0.022739*	*0.014101*
Dumpala versus Acir	0.624206	6.242061	0.624206	0.624206	0.624206

The p values below 0.05 were marked with the italic font

From Table 9, it can be observed that *p* values for unadjusted values and adjusted p values for Holm’s, Shaffer and Bergmann-Hommel offer for eliminating nine hypotheses. However, Nemenyi lets for eliminating only seven hypotheses. Based on unadjusted p values and adjusted p values for Nemenyi, Holm’s, Shaffer, and Bergmann-Hommel test, the AMSKF model revealed significantly better performance than other models.

### Application of the proposed AMSKF model to epileptic and non-epileptic EEG event classification

Two EEG events have been assigned which are epileptic and non-epileptic events. 100 non-epileptic events are collected from set A while 100 epileptic peak events from set E. Each EEG event is a segment that consists of 4097 sampling points and the duration is about 23.6 s. The best combination of peak feature and the trained NNRW classifier with 500 hidden neurons are used to perform the classification. To distinguish between epileptic and non-epileptic events, the voting method is used. The epileptic event is recognized when more than 50 peaks are identified in within an event. Whereas, the non-epileptic event is recognized when lower than 50 peaks are identified.

Table 10 demonstrates the confusion matrix of epileptic and non-epileptic event classification using the proposed AMSKF model. It can be observed that the AMSKF model obtains 98 % of total accuracy, with 100 % of the non-epileptic event rate, and 96 % of the epileptic event rate. There are four misclassifications of epileptic event.

**Table 10:** Confusion matrix of epileptic and non-epileptic event classification

Peak model	Output/desired	Result (non-epileptic event)	Result (epileptic event)	Total accuracy (%)
AMSKF	Result (non-epileptic event)	100	4	98
	Result (epileptic event)	0	96	

 The performance comparisons have been done to observe the efficiency of the proposed method. Table 11 gives the classification accuracy of this study and the existing methods on Bonn University EEG database. Referring to Table 11, the classification accuracy of this study using the NNRW method is lower than AIRS-PCA-FFT and Wavelet-ANFIS methods. However, the classification accuracy of the NNRW using AMSKF model is higher than other methods.

**Table 11:** Performance comparison of other methods

Author (year)	Method	Accuracy (%)
Proposed work (2016)	AMSKF-NNRW	98
Polat and Gunes (2008)	AIRS-PCA-FFT	100
Guler and Ubeyli (2005)	Wavelet-ANFIS	98.7
Subasi (2007)	Wavelet-MLPNN	93.6
Subasi (2007)	Wavelet-ME	95
Kannathal et al. (2005)	ANFIS	95
Guler et al. (2005)	Recurrent neural networks	96.8

**Fig. 9:**
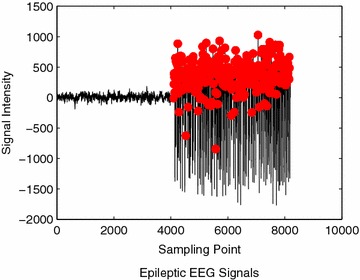
Example of epileptic event classification using record Z001 and S001

**Fig. 10:**
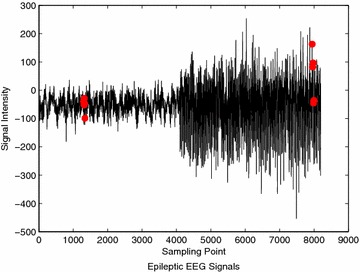
Example of misclassification of epileptic event in record Z083 and S083

An example of epileptic and non-epileptic events classification is illustrated in Fig. 9. As can be seen that, there are more than 50 peaks (red dotted) have been identified in epileptic segment (the right side) within the region from 4000 to 8000 sampling points. Figure 10 shows an example of misclassification of epileptic event in record S083. The number of detected peaks obviously can be seen is lower than 50. Consequently, the actual epileptic event is classified as non-epileptic event.

## Conclusions and future works

In this study, a new generalized peak model for EEG signals peak classification has been identified using a novel AMSKF feature selection approach. The proposed algorithm considered 11,781 peak candidate samples of real EEG data, which were collected from 30 healthy subjects instructed to direct their single eye blink, double eye blink, and horizontal eye gaze. The detection performance of the NNRW with four different peak detection models and new AMSKF model are compared. In general, the experimental results showed that the accuracy of the NNRW with new AMSKF model is better than the NNRW with other models. The statistical analysis showed that the detection performance of the NNRW with the new AMSKF model is significantly better in terms of testing accuracy compared to other models.

A published EEG database from Bonn University was selected to evaluate the proposed method and at the same time applied the relevant combination of peak features for epileptic EEG signals application. From set A and set E of the published EEG database, 20,000 peak candidate samples consist of epileptic peak and non-epileptic peak points were archived as EEG data for analysis. The major finding of this chapter is that the proposed generalized AMSKF model and NNRW classifier perform at par than the existing methods.

This study may provide a significant contribution to medical diagnostic, human–machine interface (HMI), brain-computer interface (BCI), and harmonic detection in digital and audio signal processing as these applications share a common peak detection problem. For example, an EEG peak in response to a change of horizontal eye gaze direction might be useful for patients with locked-in syndrome or other disabilities for controlling the direction of computer cursor in BCI applications. (Belkacem et al. 2014). This approach might also be translatable for EEG-based command of the movement of a robotic arm or wheelchair in HMI applications (Postelnicu et al. 2011; Ramli et al. 2015; Aziz et al. 2014).
